# Plant–Soil–Climate Interaction in Observed and Simulated Tree-Radial Growth Dynamics of Downy Birch in Permafrost

**DOI:** 10.3389/fpls.2022.780153

**Published:** 2022-05-31

**Authors:** Marina V. Fonti, Ivan I. Tychkov, Vladimir V. Shishov, Alexander V. Shashkin, Anatoly S. Prokushkin

**Affiliations:** ^1^Laboratory of Ecosystems Biogeochemistry, Institute of Ecology and Geography, Siberian Federal University, Krasnoyarsk, Russia; ^2^Forest Dynamics, Swiss Federal Institute for Forest, Snow and Landscape Research Wald, Schnee und Landschaft, Birmensdorf, Switzerland; ^3^Institute of Fundamental Biology and Biotechnology, Siberian Federal University, Krasnoyarsk, Russia; ^4^Laboratory of Complex Research of Forest Dynamics in Eurasia, Siberian Federal University, Krasnoyarsk, Russia; ^5^Environmental and Research Center, South China Botanical Garden, Chinese Academy of Sciences, Guangzhou, China; ^6^Laboratory of Tree-Ring Structure, V.N. Sukachev Institute of Forest, Siberian Branch of the Russian Academy of Sciences, Krasnoyarsk, Russia; ^7^Laboratory of Biogeochemical Cycles in Forest Ecosystems, V.N. Sukachev Institute of Forest, Siberian Branch of the Russian Academy of Sciences, Krasnoyarsk, Russia; ^8^Department of Ecology and Nature Management, Institute of Ecology and Geography, Siberian Federal University, Krasnoyarsk, Russia

**Keywords:** seasonal and annual tree-growth dynamic, temperature, precipitation, process based Vaganov-Shashkin model, VS-oscilloscope

## Abstract

Climate change projections forecast most significant impacts on high-latitude forest ecosystems. Particularly, climate warming in boreal regions should increase fire severity and shorten its return interval. These processes can change the dynamics of boreal forests as younger stands become more dominating with a shift from gymnosperm to angiosperm. However, despite angiosperm’s phenological and physiological traits have a high potential for ecophysiological and dendroclimatological studies in Siberia, they have been rarely investigated due to their short-term lifespan in comparison with gymnosperm. Modeling tree growth is a common way to understand tree growth responses to environmental changes since it allows using available experiment or field data to interpret observed climate–growth relationships based on the biological principles. In our study, we applied the process-based Vaganov–Shashkin (VS) model of tree-ring growth *via* a parameterization approach VS-oscilloscope for the first time to an angiosperm tree species (*Betula pubescens* Ehrh.) from continuous permafrost terrain to understand its tree-radial growth dynamic. The parameterization of the VS model provided highly significant positive correlations (*p* < 0.05) between the simulated growth curve and initial tree-ring chronologies for the period 1971–2011 and displayed the average duration of the growing season and intra-seasonal key limiting factors for xylem formation. Modeled result can be valid at the regional scale for remote birch stands, whereas, justification of the local non-climatic input data of the model provided precise site-specific tree growth dynamic and their substantiated responses to driving factors.

## Introduction

The global increase in surface air temperature that started in early twentieth century (IPCCV, 2013) and projected future climate changes suggest that forest ecosystems at high-latitude regions will likely be subjected to significant impacts (Serreze et al., 2000; Sugimoto et al., 2002; Delisle, 2007; Sanderson et al., 2011). Northern forest ecosystems of the boreal zone will be particularly exposed to warming and changes in precipitation regimes causing permafrost degradation (Osterkamp and Romanovsky, 1999; Schuur et al., 2008). At the same time, climate change is altering the fire regime in northern high-latitude regions, increasing annual burned area in Alaska (Kasischke and Turetsky, 2006; Kasischke et al., 2010), Canada (Gillet et al., 2004; Hanes et al., 2019), and Eurasia (Hayes et al., 2011; Kharuk et al., 2013; Shuman et al., 2017; Novenko et al., 2022).

Forest fires have a strong influence on forest dynamics, as well as on the structure and functioning of forest ecosystems. The reason for such a crucial impact is that the thermal balance of each element (plant community, microorganisms, soil properties, and structure, etc.) in continuous permafrost terrain is very weak due to low resilience and simple structure (Pozdnyakov, 1986; Holden et al., 2016). The heat transfer to subsoil during a fire decreased albedo and lower soil insulation after a fire result in a deepening of the active layer and thawing near-surface permafrost (Johnstone et al., 2010; Kharuk et al., 2011; Nossov et al., 2013; Kirdyanov et al., 2020). Fire affects carbon balance directly by burning vegetation and surface organic material and indirectly by influencing post-fire vegetation composition and soil hydrothermal and edaphic conditions (Prokushkin et al., 2000; Harden et al., 2006). Although boreal ecosystems have been exposed to periodic fires, increased fire frequency due to climate change has transformed the age (old to young) and composition (gymnosperm to angiosperm) of the vegetation and its recovery (Czimczik et al., 2006; Moser et al., 2010; Tautenhahn et al., 2016).

Despite the wide range of the dendroecological and climatological studies in the northern hemisphere, research has mainly been focused on tree-ring width chronologies of conifer species due to their distribution, longevity, and capability to fix long-term climatic signals [e.g., Vaganov et al. (2000); Esper et al. (2002), Briffa et al. (2008); Nikolaev et al. (2009), Churakova Sidorova et al. (2020), etc.]. Much less attention was given to angiosperm species (Goldblum and Rigg, 2005; Schmidt et al., 2006; Sano et al., 2010; Babushkina et al., 2019). Angiosperms have different phenological and physiological features than gymnosperm. For example, they differ in the effects of phenology on their productivity, growth allometry, sensitivity to competition, hydraulic safety margins, sensitivity of stomatal conductance to vapor-pressure deficit (VPD), xylem recovery capacity or the rate of carbon transfer (Carnicer et al., 2013), and resilience strategies (DeSoto et al., 2020). As a result, their capability to fix environmental signals in tree ring during the growing season differs from conifer trees and has a high potential for an ecophysiological and dendroecological studies. Experiments and field studies on long-term and large-scale processes for angiosperm species of the permafrost zone in Northern Russia are rare, except for some analyses of shrubs (Forbes et al., 2010; Blok et al., 2011; Arefyev, 2015) showing strong climate–growth relationship. Genus *Betula* L. covers a wide area and different species of birch are an essential component of northern ecosystems (Kullman, 1993; Sano et al., 2010; Koropachinskii, 2013; Drobyshev et al., 2014; Bandekar and Odland, 2017; Harr et al., 2021). Hybridization and introgression of birch species (e.g., *Betula pendula*, *Betula pubescens*, and *Betula nana*) are common if their distribution overlaps (Palme et al., 2004). In Northern Russia, *B. pendula* Roth. and *B. pubescens* Ehrh. are the main deciduous angiosperm tree species with significant ecological, economical, and landscape value (Vetchinnikova, 2004; Zyryanova et al., 2010). They mainly share the territory, but downy birch (*B. pubescens* Ehrh.) is characterized by higher plasticity and expands far to the north (Vetchinnikova, 2004).

To understand the tree growth pattern of birch, simulation models can be used to evaluate plant–soil–climate interaction. Process-based models have been used for several decades in dendroclimatology, but it is only recently that their application has led to significant progress in modeling tree growth as a function of climate and to reconstruct past phenology of tree-ring growth (Guiot et al., 2014; Hartmann et al., 2017; Yang et al., 2017; Anchukaitis et al., 2020; Shishov et al., 2021). The Vaganov–Shashkin (VS) model of tree-ring formation was a pioneer in representing daily xylem growth principles by explicitly incorporating multivariate environmental controls on tree-ring growth (Shashkin and Vaganov, 1993; Vaganov, 1996; Vaganov et al., 2006).

In this study, for the first time, we apply the VS-model of tree-ring growth (Vaganov et al., 2006) to understand tree-radial growth dynamic of angiosperm tree species growing on permafrost. The parameterization of the model *via* VS-oscilloscope (Shishov et al., 2016) was applied with a seasonal soil-thawing block. We hypothesize (1) that birch (*Betula pubescens* Ehrh.) growth rate can be simulated based on the high relation between tree growth and climate conditions in Siberian North; (2) that modeled results at the regional scale will be valid across stands due to highly coherent tree growth responses over the vast northern territories [e.g., as for conifers, Vaganov et al. (2000) and Esper et al. (2002)]; (3) the model is sensitive enough to account for non-climatic site-specific differences within the region.

## Materials and Methods

### Study Area

The study area is located in northern taiga of central Siberia (Russia, 64°18′ N, 100°11′ E) and is characterized by continental climate with annual air temperature –9°C and the annual precipitation 370 mm (data from the Tura meteorological station of the Russian Research Institute of Hydrometeorological Information Data Base for the period 1936–2012).^1^ The growing season based on xylogenesis observations of *Larix gmelinii* (Rupr.) Rupr.—a main tree species covering about 82% of the studied territory—usually starts at the end of May and ceases in late August or early September (Bryukhanova et al., 2013).

**FIGURE 1:**
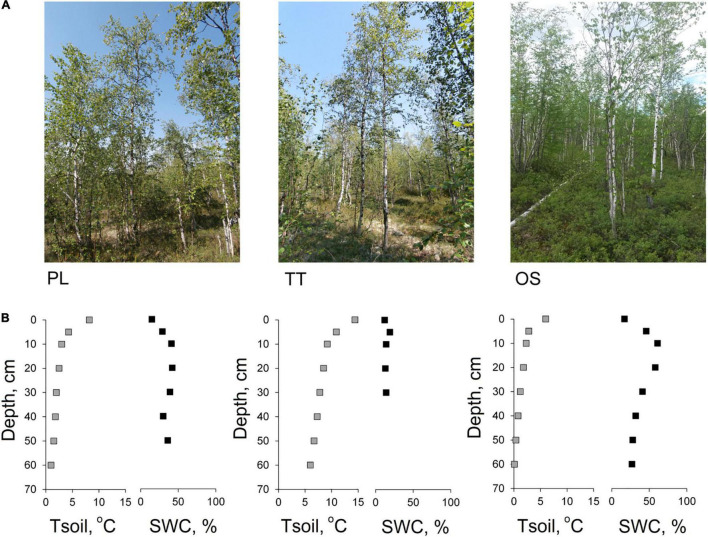
Photograph of the fire-origin birch stands (PL—110 years old, TT—114 years old, OS—54 years old) **(A)**, and 60-cm-depth profiles of soil temperature (Tsoil—gray square) and gravimetric soil water content (SWC—black square) obtained in the middle of the growing season **(B)**.

*Betula pubescens* Ehrh. is distributed over the upland flat surfaces of traps at altitudes up to 700 m above sea level and spreads on 11% of territory (Korets et al., 2016). A number of three sites (PL, OT, and OS) were chosen 20 km apart on flat areas of Central Siberian traps within Syverma Plateau and represented by birch forests (*B. pubescens* Ehrh.) mixed with larch (*L. gmelinii* (Rupr.) Rupr.) and spruce (*Picea obovata* Ledeb.) as a post-fire succession stage with even age structure (Figure 1A). Dominant trees of birch at all studied sites were characterized by similar height and stem diameter at the breast height (Table 1). The understory and ground vegetation mainly consists of undershrubs [e.g., *Alnus alnobetula* subsp. *fruticosa* (Rupr.), *Ledum palustre* L.], mosses [*Pleurozium schreberi* (Brid.) Mit., *Aulacomnium palustre* (Hedw.) Schwaegr.], and lichens (*Cladina* spp., *Cetraria* spp.).

**TABLE 1:** Description of the studied stands.

Site	Location	Stand density, trees/ha	Mean tree height, m	Mean tree diameter at the breast height, cm	Mean tree height of analyzed trees, m	Mean tree diameter at the breast height of analyzed trees, cm	Tree age, years
PL	64°18′N, 100°25′E, 590 m a.s.l.	8,000	4.0	3.9	8.0	10.0	110
TT	64°12′N, 100°26′E, 80 m a.s.l.	6,450	4.3	4.0	8.0	9.3	114
OS	64°17′N, 100°08′E, 388 m a.s.l.	4,600	4.9	4.3	7.9	8.0	54

Temporary test plots (radius of 15 m) were chosen within the studied stands, the height and diameter at breast height of all trees were measured, and the stand density was estimated.

To determine tree growth of downy birch, we have collected wood cores of 5 mm in diameter from stems at breast height for 20–22 dominant trees per site. The cores were collected perpendicular to the stem axis avoiding reaction wood.

### Soil Conditions

The active soil layer depth (ALT) and microrelief were measured along a 10-m transect with a 1-m step at the beginning of August 2012. In parallel, samples of the organic layer and moss-lichen stratum (100 cm^2^, *n* = 11) were taken to measure the plot mean stock of the organic matter accumulated on the surface of the mineral soil (Table 2). According to observations, at the date of measurements, the active soil layer depth reached 70–80% of the maximum observed at the end of September—beginning of October. In areas where the active layer was more than 1.2 m and digging was impossible, the permafrost depth was estimated using a linear regression model of the soil temperature in the mineral soil against the depths (Zhou et al., 2019).

**TABLE 2:** Site soil properties.

Site	Microtopography, cm	Active soil layer thickness, cm*	Gravimetric soil water content at a depth of 5 cm, %*	Soil temperature at a depth of 5 cm, °C*	Organic matter stock (mosses + organic horizon), kg/m^2^
PL	<5	85	29	2.8	2190.7 ± 268
TT	10	152	19	10.9	1493.1 ± 455
OS	>10	62	46	4.3	2881.8 ± 422

*Measurements in August 2012.

To obtain general soil characteristics, we dig a soil pit to the parent rock (basalts) in the central part of the plot. The soils of all studied sites were well-drained, formed by coarse-grained material, thin (the depth of the profile to the parent rock is 40–60 cm), and belong to the Spodic Cryosols [AO(O)—Bf(Bh,f)—C] (IUSS Working Group WRB, 2015). The soil temperature was measured in every soil pit with a step of 5 cm with a Hanna HI 935005 soil thermometer in the horizontal direction of the soil section (probe length—220 mm). Soil moisture was determined in soil samples taken at 5-cm interval by gravimetric method (Figure 1B).

### Dendrochronological Measurements and Statistical Analysis

The measurements of birch annual tree-ring width (TRW) for three studied sites (Fonti and Prokushkin, 2021) were performed using a LINTAB measuring table with 0.01 mm precision combined with the program TSAP (Rinntech, Heidelberg, Germany). The TRW of birch trees from PL site was measured on 20-μm-thick microsections prepared with a sledge microtome (Heidelberg, Reichert, Germany) and stained with blue methylene to facilitate the identification of very narrow rings.

Obtained time series was visually cross-dated and dating quality verified using the program COFECHA (Holmes, 2001). To assess climate–growth relationships, raw tree-ring width series were standardized to remove non-climatic trends (Cook and Kairiukstis, 1990). Negative exponential function was applied as a detrending method. Time series for individual trees was averaged to obtain the site chronologies. Regional tree-ring width chronology (REG) was calculated based on all collected trees from the three study sites.

Growth responses to climate were evaluated by calculating Pearson’s correlation between regional and site chronologies and monthly temperature and precipitation from the Tura meteorological station for the common period from 1971 to 2011. Analysis was performed for each month from May of the previous year to September of the current year. Moving-window correlation was calculated between TRW series and climatic factors (20-day window with 5-day step) to identify growing season periods with a higher climatic signal.

### Model Description

We used the process-based VS model (Vaganov et al., 2006) *via* a parametrization approach of the VS-oscilloscope (Shishov et al., 2016)^2^ to simulate *B. pubescens* tree-ring growth as a function of climatic conditions (day length, daily temperature, and precipitation) and soil (seasonal soil thawing depth) parameters. Beginning of the growing season was defined as the period with temperature above 5°C (Tmin) which only initiate after a certain period with cumulative temperature above a threshold (Tbeg). The end of growth occurs when the integral growth rate (Equation 1) of the tree ring falls below a critical threshold (critical growth rate Vcr) (Tychkov et al., 2019; Table 3).

**TABLE 3:** Vaganov-Shashkin-model input parameters for the studied period 1971–2011 [for three sites PL, OS, and TT, and for the regional chronology (REG)].

Parameter	Description (Units)	REG	Local PL	Local OS	Local TT
*T*_*min*_	Minimum temperature for tree growth (°C)	5	5	5	5
*T*_*opt*1_	Lower end of range of optimal temperatures (°C)	19	19	19	19
*T*_*opt*2_	Upper end of range of optimal temperatures (°C)	23	23	23	23
*T*_*max*_	Maximum temperature for tree growth (°C)	25	25	25	25
*W*_*min*_	Minimum soil moisture for tree growth, relative to saturated soil (volume/volume ratio)	0.015	0.047	0.002	0.025
*W*_*opt*1_	Lower end of range of optimal soil moistures (volume/volume ratio)	0.275	0.200	0.250	0.100
*W*_*opt*2_	Upper end of range of optimal soil moistures (volume/volume ratio)	0.575	0.525	0.350	0.575
*W*_*max*_	Maximum soil moisture for tree growth (volume/volume ratio)	0.575	0.525	0.400	0.600
*l*_*r*_	Depth of root system (mm)	300	250	450	250
*P*_*max*_	Maximum daily precipitation for saturated soil (mm/day)	55	38	43	57
*C*_1_	Fraction of precipitation penetrating soil (not caught by crown) (rel. unit)	0.52	0.60	0.53	0.55
*C*_2_	First coefficient for calculation of transpiration (mm/day)	0.185	0.072	0.195	0.165
*C*_3_	Second coefficient for calculation of transpiration (1/°C)	0.105	0.055	0.125	0.135
Λ	Coefficient for water drainage from soil (rel.unit)	0.006	0.006	0.006	0.006
*T*_*beg*_	Temperature sum determining growth start (°C)	91	92	93	97
*V*_*cr*_	Minimum cambial cell growth rate (no units)	0.02	0.02	0.02	0.02
*T*_*melt*_	Sum of temperatures in 10 days for beginning of soil thawing (°C)	40	40	40	40
*Sm1*	The first coefficient of soil defrosting (mm/°C)	9	9	9	9
*Sm2*	The second coefficient of soil defrosting (1/day)	0.006	0.006	0.007	0.006

The VS-model is based on several assumptions. First, the main target of external influence is the cambial zone, the zone of actively dividing cells. The external factors influence growth, division, and differentiation of cambial cells. Second, the main external factors that affect cambial cell growth are temperature, soil moisture, and daily solar radiation. The model estimates a daily water balance based on accumulated precipitation into the soil, transpiration, and drainage (Thornthwaite and Mather, 1955). Daily solar irradiance is determined as a function of test polygon latitude, and day of the year (Gates, 1980). Third, in growth rate calculation, the principle of limiting factors is used, i.e., growth rate at a certain interval (day) of a season cannot be higher than allowed by the factor that is most limiting. Using the model, we determined the principal factors driving growth at the daily scale based on the most limiting partial growth rates induced by temperature (heat limited and cold limited) and soil moisture (moist limited and drought limited). If both (soil moisture and temperature) daily rates were equal to 1, we considered that tree-ring growth had occurred in optimal climatic conditions [e.g., Tumajer et al. (2021a; 2021b)]. The integral growth rate of tree rings *Vext*(t) and external factors of the day t are described by the equation (Vaganov et al., 2006; Shishov et al., 2021):


(1) $$V\,e\,x\,t\,\left(t\right)=V_{I}\,\left(t\right)\,\min\,\left[V_{T}\,\left(\text{t}\right),V_{W}\,\left(\text{t}\right)\right]$$

where *V*_*I*_(t), *V*_*T*_(t), and *V*_*W*_(t) are the partial growth rates dependent on solar radiation (day length) (I), temperature (T), and soil moisture (W), respectively.

The VS model conception is based on the suggestion that seasonal tree-ring growth is exclusively forced by the common climatic signal. Therefore, other factors that influence tree-ring growth [age-depending trends, trees competition, abrupt disturbances (fires and insect outbreaks), etc.] can be considered as a noise. To avoid the influence of non-climatic factors, we used standardized time series.

The principal goal of the model parameterization is to get a best fit of the simulated tree-ring curves to the observed tree-ring chronologies by selecting parameters whose values are unknown or unavailable. At the same time, the selected values should follow the biological principles of growth and correspond to the field observations of the studied regions. The solution of this task by direct mathematical optimization of multidimensional parameter space is problematic taking into account a high probability to reach local optimum generating artificial decisions (Evans et al., 2006; Tolwinski-Ward et al., 2013). Not only higher Pearson’s correlation coefficient (R), but also synchronicity [Gleichläufigkeit (Glk), Eckstein and Bauch (1969)] and root mean square error (RMSE) were considered as the indicator of the strength of the common signal between observed and simulated chronologies.

To validate the model results, the whole studied period from 1971 to 2011 was divided into two independent intervals: calibration (1991–2011) where the model was parameterized and verification (1971–1990) to check a forecast power of the model.

Trees growing on permafrost may use two water sources—atmospheric precipitation and water from the thawing of upper permafrost, the active layer. The process of thawing and water content in the active layer, some of which come in the current year and some of which are retained from the previous year, is taken into account only for high-latitude regions. The rate of thawing is described as proportional to temperature (Kuzmin, 1961) and exponentially decreases with the increasing thickness of the thawing layer. Since the study sites are located in the permafrost zone, the soil-thawing block was applied for tree growth simulation. The main input parameters used in the VS model are listed in Table 3.

## Results

### Statistical Parameters of Tree-Ring Width Chronologies and Climate–Growth Relationship

Tree-ring width analysis has shown that the OS site is almost 2-fold younger (54 vs. 110 years) than PL and TT (Table 4). Raw site chronologies showed well-pronounced growth patterns. TRW at PL increased since the 1990s, and this tendency is obvious for all the trees from this site. Opposite to that, the mean TRW chronologies at OS and TT sites showed decreasing trend for the same period, which was observed for most of the individual series with some exceptions (*n* = 2) for TT.

**TABLE 4:** Statistical parameters of tree-ring width chronologies.

Site	Number of trees	Period	Mean tree-ring width ± se*, mm	Correlation radii vs. mean	Standard deviation	Mean sensitivity	1st order autocorrelation
PL	22	1902–2011	0.41 ± 0.01	0.663	0.32	0.35	0.28
TT	20	1901–2014	0.37 ± 0.01	0.580	0.25	0.26	0.25
OS	20	1961–2014	0.68 ± 0.01	0.770	0.31	0.25	0.41

*se—standard error.

The statistical parameters of the chronologies also recorded considerable similarity of TRW (Table 4). The mean radial growth at PL and TT is lower than at OS. Trees from OS site have a growth variation that could be related not only to environmental conditions but also to the stand age.

Strong common signals between individual trees at each site (mean inter-series correlation of 0.66, 0.77, and 0.58 for PL, OS, and TT, respectively) indicate the dominance of a common factor on tree growth. The first-order autocorrelation explained less than 10% of TRW variability at PL and TT, and up to 16% for OS. Indexed site chronologies showed similar year-to-year variations in radial growth for the period 1971–2011 (*R*_PL–OS_ = 0.60, *R*_OS–TT_ = 0.60, *R*_TT–PL_ = 0.57, *p* < 0.05). Regional chronology is also correlated significantly with site chronologies (*R*_REG–PL_ = 0.84, *R*_REG–OS_ = 0.86, *R*_REG–TT_ = 0.88, *p* < 0.05).

**FIGURE 2:**
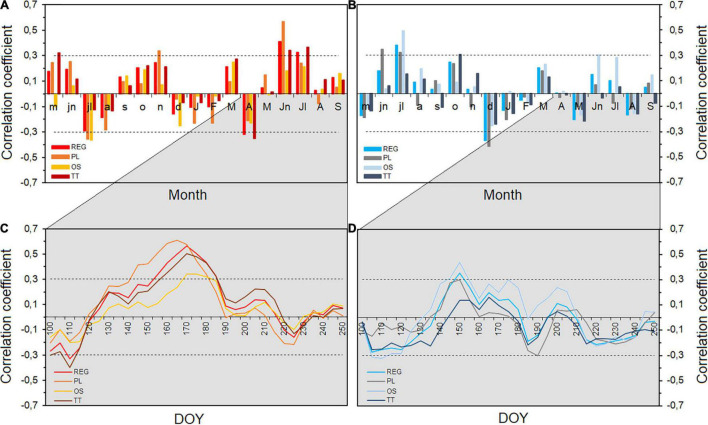
**(A)** Monthly correlation of tree-ring width standardized regional and site chronologies (REG, PL, TT, and OS) with temperature and **(B)** with precipitation from May of the previous year to September of current year for the common period 1971–2011. Moving correlation from April to September [100–250 days of the year (DOY)] (window = 20 days and step = 5 days) with temperature **(C)** and precipitation **(D)**. Horizontal dashed lines show significant correlations at *p* < 0.05.

Results of the dendroclimatic analysis with monthly temperature and precipitation for the period 1971–2011 indicate a positive growth response to June and July temperature with the regional TRW chronology (*R* = 0.40 and 0.33, respectively, *p* < 0.05) and the TT site (*R* = 0.34 for June and 0.37 for July, *p* < 0.05). Tree-ring growth at PL site was instead mostly defined by only the temperature of June (*R* = 0.57, *p* < 0.05), whereas the OS site did not show significant correlation between tree-ring growth and summer temperatures (Figure 2A). Precipitation of the current summer significantly affected the TRW at OS site only (correlation with June precipitation is 0.30, *p* < 0.05) (Figure 2B). Interesting to notice is the positive relation between tree growth and previous year summer precipitation for the regional chronology (*R* = 0.38 for July, *p* < 0.05), the PL site (*R* = 0.35 and 0.32 for June and July, *p* < 0.05), and the OS site (*R* = 0.49 for July, *p* < 0.05). Negative correlation (*R* = –0.36, *p* < 0.05) was observed for TRW and July temperature of previous year for both PL and OS. No significant correlation for TT site with the previous year climate has been found.

To compare the timing of the climatic response over the growing season among sites, we calculated climate–growth relationships over a 20-day window moving across the growing season (Figure 2C). This detailed analysis fully confirmed and complemented previous results showing a consistent strong summer temperature signal in the regional and the two PL and TT site chronologies. The signal started to be significant earlier in mid-May for PL site, approximately 2 weeks later in TT site and almost with 1-month delay (started at the end of June) for OS site. Regional TRW chronology showed a significant correlation with temperature from the beginning of June until end of July [from 155 to 205 day of the year (DOY)]. The highest correlation (*R* = 0.61, *p* < 0.05) was obtained between TRW of PL-site and 20-day period at the end of June (165–185 DOY). Less coherent was the climatic signal to precipitation, which strength, timing, and duration varied depending on the sites, with more significant impact on tree growth at the OS site (Figure 2D).

### Soil Hydrothermal Conditions and Its Effect on Tree-Radial Growth

The OS site displayed the shallowest active soil layer thickness, which thawed to a maximum of 62 cm depth compared to other studied sites (ALT_PL_ = 85 cm, ALT_TT_ = 152 cm). At the same time, soil water content at the OS site was the highest and varied between 16 and 68% at the depth between 0 and 30 cm (and further was slightly decreasing with soil depth) (Figure 1B). The TT site demonstrated the shallowest soil profile as the parent rock was observed already below the 40 cm depth and driest (SWC up to 19%) in comparison with other sites. In opposite, the site has the highest soil temperature, in which average was 6 and 7.2°C higher compared to PL and OS, respectively. Despite the similar age of the stands at PL and TT sites, the surface organic layer accumulated on the mineral soil differed among the plots [almost two times as much on PL (2,190.7 kg/m^2^) as on TT (1,493.1 kg/m^2^)]. The OS showed the highest value (2,881.8 kg/m^2^). The trees growing at OS site with higher soil water content and cumulated organic material were characterized by wider tree-ring width in comparison with other studied sites.

### Simulated Regional Birch Tree Growth, Duration of the Growing Season, and Intra-Seasonal Driving Factors

**FIGURE 3:**
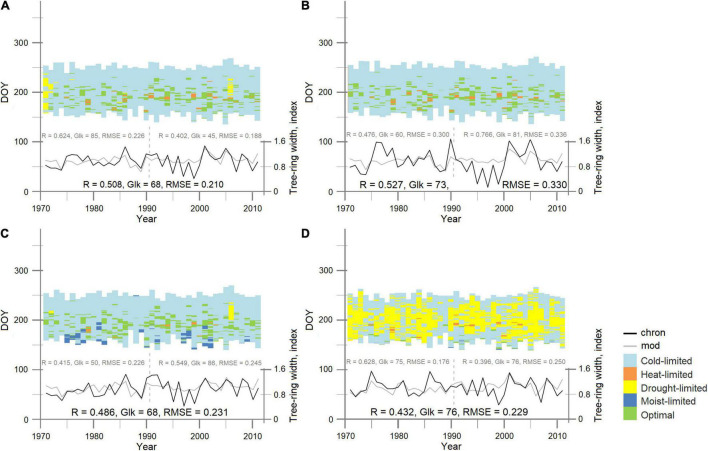
Observed (chron—black solid line) and simulated (mod—gray solid line) tree-ring width regional **(A)** and site birch chronologies **(B)** PL, **(C)** TT, **(D)** OS, for the period 1971–2011. The vertical dashed line separates the calibration period (1991–2011) from the verification period (1971–1990). Pearson’s correlation coefficient (R), synchronicity [Gleichläufigkeit (Glk)], and root mean square error (RMSE) shown for each period/site. Each chart presents the site-specific integral growth rates for each day of the growing seasons. Colors depict the driving climatic parameters. Input model parameters for each simulated curve are presented in Table 3.

Based on the estimated input model parameters (REG, Table 3) highly significant positive correlation was obtained between the regional chronology and estimated growth curve (*R* = 0.51, *p* < 0.05) for the period 1971–2011 (*n* = 41 years) (Figure 3A). Output data of the model simulation showed that the duration of the growing season for the studied period was 98 ± 10 days (mean ± standard deviation) with the shortest in 1989 (76 days) and the longest in 2005 (131 days) and influenced positively on tree-ring growth (*R* = 0.46, *p* < 0.05). At the same time, TRW was significantly correlated with the beginning of the vegetation period (*p* < 0.05), and not significant with its cessation, indicating that the earlier start of the growing season promoted wider tree ring, which was more obvious for the last decade with the observed increase in May temperature.

At the regional level, annual birch tree-ring growth was mostly limited by cold temperature. In 1971, 1972, 1985, 1986, and 2006, the model output showed several days during the growing season where growth was limited by drought. Occasional occurrence of days with heat limitation was observed for 20 vegetation periods out of 41 (full period of analysis). The increasing trend in the frequency of days with optimal growth conditions facilitated annual growth (*R* = 0.44, *p* < 0.05) (Figure 3A).

### Justification of the Site Local Input Data of the Vaganov-Shashkin Model

Application of the regional input parameters to the individual site chronologies showed a significant correlation (*p* < 0.05) for remote birch stands of PL, OS, and TT due to the similarity of climatic conditions over the vast northern territories. To obtain more precise simulated individual tree-growth dynamic for each stand, the biologically justified local soil-related input model parameters, which influenced soil moisture content and consumption during the growing season and modified climatic signal at the stand level under changes of limiting factors, were applied. Since trees from OS site were younger and presented an earlier fire successional stage with a deeper active soil layer in comparison with PL and TT sites, they have a deeper root system. According to that, in the input data of the model, the depth of roots was changed from 30 cm of REG to 45 cm of OS (Table 3, *Local OS*) and high-up the correlation between the initial OS chronology and estimated growth curve from *R* = 0.37 to *R* = 0.43 (*p* < 0.05) for the period 1971–2011. Such parameterization not only allowed to improve the estimated tree-ring width curve, but also helped to precisely clarify the influence of the observed driving factors during the growing season (Figure 3). Since the age of the stands at the TT and PL sites was similar, but soil temperature and soil moisture were different (Figure 1 and Table 2), we assumed that the transpiration rate could be different (Table 2, *Local TT* and *Local PL*). With the adjusted model parameters, correlation between measured and calculated TT-tree-ring width chronologies increased from *R* = 0.46 to *R* = 0.49 (*p* < 0.05) and from *R* = 0.47 to *R* = 0.53 (*p* < 0.05) to PL site. Based on the local parameterization of the models input dataset, it was possible to explain site differences in climate response with daily resolution (Figures 3B–D). It was clearly shown that tree growth at the OS site was much stronger dependent on soil moisture content (limitation by drought) during the growing season (up to 44%) in comparison with PL and TT sites (up to 5% only). Such results support the climate–growth correlation, which is obtained between TRW and precipitation (Figure 2D). As it was mentioned earlier, summer precipitation of the current and previous summer positively affects TRW_OS_. Correlation analysis of tree-ring width and daily limiting factors (expressed as a percentage of the total number of factor-days per season) shows that tree growth at the PL and TT sites reduced during the years with long cold phases.

### Integral Growth Rates

**FIGURE 4:**
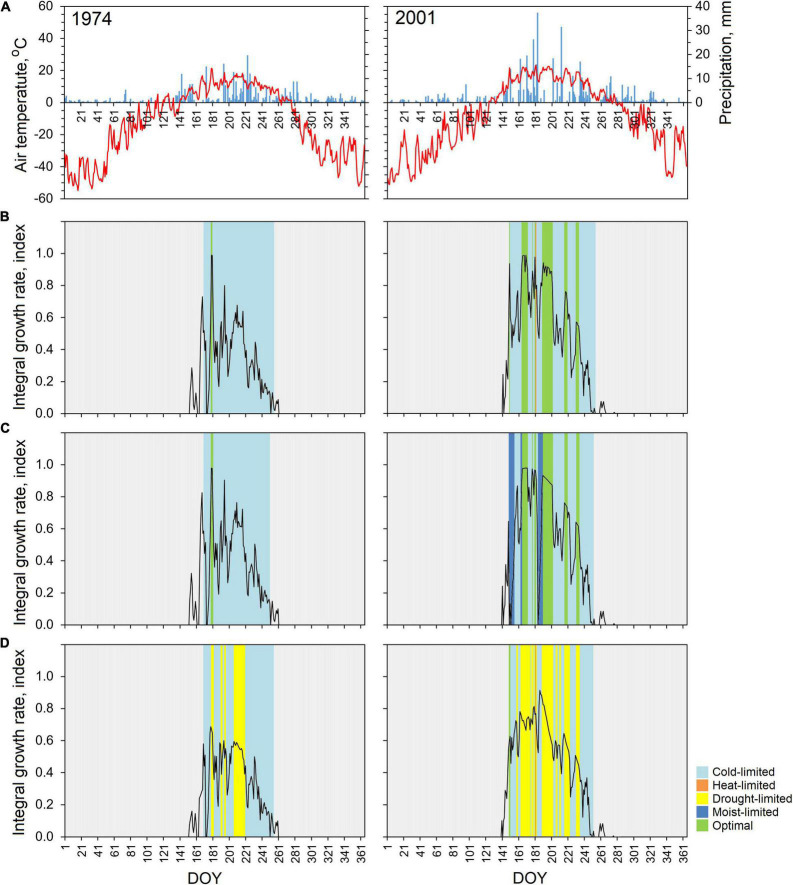
Daily air temperature (red line) and amount of precipitation (blue column) during 1974 and 2001 **(A)**, and daily integral growth rate of *Betula pubescens*: **(B)** PL, **(C)** TT, and **(D)** OS. Colors depict the driving climatic parameters.

Based on the climatic data analysis on June–July, two extreme years, the coldest 1974 and the warmest 2001 (with air temperature 11.4 and 17.5°C, and amount of precipitation 111.9 and 225.8 mm, respectively), were chosen to understand how trees at local and regional level responded to these contrasting weather conditions at the intra-annual scale (Figure 4). For all the studied stands, dramatic decrease in TRW was observed in 1974 [0.10 ± 0.07 mm for PL, 0.17 ± 0.08 mm for TT, and 0.69 ± 0.22 mm for OS (mean ± standard deviation)], whereas, in 2001, radial growth was highest for the last decade (TRW_PL_ = 0.47 ± 0.19, TRW_TT_ = 0.37 ± 0.27, TRW_OS_ = 0.92 ± 0.27).

Tree growth in 1974 was mainly sensitive to air temperature, when its low values in couple with an average amount of precipitation throughout vegetation period affected the integrated growth function and results in narrow observed and simulated TRW. Low spring temperatures caused a shift of the beginning of the growing season for almost 3 weeks (170 DOY) in comparison with average, with the mean duration of the growing season of 86 days.

In 2001, the beginning of the growth season started at the end of May (143 day of the year) and lasted 105 days (Figures 4B–D), and the growth function remained at high level until the middle of June, due to positive precipitation to evapotranspiration relation. High summer temperature with significant amount of precipitation stimulated tree growth at all studied sites. As a result, a relatively wide ring was formed during this year. Information on daily driving factors was of particular interest. If for coldest 1974, the differences between TT and PL were almost invisible, the growing season of 2001 was characterized by site-specific pattern related to the difference in limiting factors among the sites clarifying why trees at the OS site showed the strong soil moisture effect (drought-limit) on tree-ring growth for both years.

## Discussion

It is widely known that the local and micro-site growing conditions (Lange et al., 2018; Gurskaya et al., 2021; Hartl et al., 2021), especially hydrothermal soil conditions in the permafrost zone (Nikolaev et al., 2009; Kirdyanov et al., 2013; Bryukhanova et al., 2015; Prokushkin et al., 2018), might significantly affect tree growth. This study showed that the application of the VS-model makes it possible to account for specific soil-related conditions in deciduous (angiosperm) stands (by modifying model input parameters accordingly to biological principles of growth specific to the site), allowing to improve the correlation between simulated and observed tree-ring width.

### Regional Characteristics of Birch Climate–Growth Relationship

The climate–growth analysis showed that at the regional scale growth of downy birch is highly correlated with June–July temperature (*p* < 0.05). Additional analysis (Fonti and Prokushkin, 2021) based on moving-window correlation (30-year window with a 1-year shifting step) also confirmed that this relation was relatively seasonally stable over the entire 1936–2012 period (i.e., the period covered by meteorological observations) but increasing in strength in the recent decades (from 1970 to 2012). At the same time, it should be noted that the monthly climate–growth relationship of the three studied sites (PL, TT, and OS) showed significant differences in correlation between TRW and temperature or precipitation, due to signal modification caused by local differences in soil and stand conditions. Analysis performed on single climatic data excludes the possibility to account for such variability.

Since the studied sites were equally undergoing the harsh climatic conditions typical of the high-latitude domain of Siberia, we hypothesized that the birch growth rate could be simulated only considering the high correlation between tree growth and climate conditions. In line with this assumption, obtained coefficients of correlation and synchronicity between indexed and simulated TRW chronologies were in the range of values achieved for other studies on coniferous species in Siberia (Vaganov et al., 2006; Shishov et al., 2016; Fonti et al., 2021). Thanks to the application of the VS-model in daily resolution, we could show that soil moisture (both drought and moist) also played an important role in determining the monthly growth at studied sites (Figure 3). For example, soil moisture affected tree-ring width during 5% of the vegetation period at TT (1% drought and 4% moist limitation) and up to 46% at the OS site.

The most extreme growing seasons, determined as a ratio of the average June–July air temperature to their sum of precipitation, affected differently the radial growth of downy birch (Fonti and Prokushkin, 2021). First, an increase in TRW was observed for all studied sites during the most favorable (warm and wet) conditions, and, second, significant differences were revealed on tree growth, when low air temperatures were more crucial for trees in the PL and TT, whereas, water availability was the main driving factor for trees in OS site.

Earlier results of the impact of climatic parameters on tree-ring growth of coniferous species in permafrost zone (Nikolaev et al., 2009; Kirdyanov et al., 2013; Shishov et al., 2016; Fonti et al., 2019) showed to some extent a common pattern with the newly obtained birch TRW signal since the studied stands are equally located in a strong temperature limiting environment. However, different characters of moisture effect on stem seasonal development were observed on both, the strength of the correlation and its seasonal duration, when these correlations are statistically significant (*p* < 0.05) over the growing season. This difference in responses to soil moisture might be generally explained by various drought tolerances of the species, confirming the different demands of larch, pine, spruce, and birch to soil moisture.

Comparison of the obtained results for the Siberian permafrost zone with the study of birch in Northern Norway (Harr et al., 2021) showed agreement in the positive growth response to June air temperature but difference regarding the May temperature response. This might be due to the relatively late start of the growing season in Northern Siberia, when the earliest period with significant (*p* < 0.05) coefficients of correlation between TRW and air temperature occurred between 145–165 DOY (late May–early June). Overall, the duration of the growing season was shorter. The growth of the *Betula ssp.* stands studied in non-permafrost areas from Fennoscandia and Canada, where the climate is milder with almost no temperature limiting growth, showed differing results mainly characterized by strongest response to available moisture, soil properties, and/or strong winds (Drobyshev et al., 2014; Bandekar and Odland, 2017).

### Soil Conditions as a Modified Factors of Tree Growth in Permafrost

As mentioned earlier, soil conditions played an important modifying role in tree-ring climate signal. Taking into account the fact that birch stands in permafrost zone have a pyrogenic origin (Pozdnyakov, 1986; Zyryanova et al., 2010), all studied sites (PL, TT, and OS) were in their late fire succession stage. However, according to their age (half as young at OS, as at PL and TT), it was possible to assume that trees have a different depth of their root system due to post-fire time-related aggradation of permafrost. According to Knorre et al. (2009) and Kirdyanov et al. (2020), rising permafrost table in larch forest of the studied territory reaches its initial pre-fire value approximately in 70–80 years. In line with this data, the input VS model parameter *Lr* (depth of root system in mm, Table 3) was adjusted accordingly at the local and regional scale and significantly improved simulation output. The hydrothermal regime (soil moisture and soil temperature), especially in root-inhabited soil horizon, also affected tree growth at local scale. Less direct but strong impact on tree growth showed the thickness of the insulating organic layer with a living ground cover vegetation (Knorre et al., 2019), controlling the soil water and temperature regime. The tree growth in the OS site was influenced by a set of soil parameters different from the other two stands (soil temperature, soil moisture, soil organic layer stock, etc.), which led to an almost 2-fold increase in TRW, regardless of the cambial age of the trees.

In the development of birch stands and the modification of the climatic signal recorded in the tree-ring width, it is worth noting the likely role of cryogenic microrelief. A number of three studied sites differ by the severity of the microrelief at the local scale. An increase of the mosaic (permafrost heaving) at the OS might have a significant effect on the tree-individual growth. Such a local impact of the hydrothermal soil conditions on tree growth was already noted earlier for the *L. gmelinii* (Rupr.) Rupr. in Northern Siberia (Bryukhanova et al., 2015).

Finding such a great effect of soil conditions on birch tree growth, more detailed and regular observations of soil parameters in remote regions of northern hemisphere without anthropogenic impact are needed. This will not only improve our understanding of the processes occurring in the permafrost zone, but also allow using the data obtained as input parameters for modeling of tree growth and woody biomass. This will open up new perspectives for more accurate and reliable forecast of the adaptation of woody plants to changing environmental conditions at the local, regional, and global scale.

## Conclusion

The process-based VS-model of tree-ring growth *via* a parametrization approach VS-oscilloscope successfully reproduced birch radial growth in continuous permafrost terrain of Central Siberia as a function of climate. The chronologies simulated by the model are correlated strongly with the regional chronology (REG) and showed significant but differing correlations at each of the tree sites (PL, OS, and TT), which have been chosen for the verification of the model at the local scale. The differences in climatic responses at the different sites (modified by the minimum soil moisture effect in PL, and maximum—in OS) were successfully attributed to changes in local soil condition by modifying soil-related model input data. Such modifications not only improved the correlation and synchronicity (Glk) between simulated and initial ring-width chronologies, but also contributed to clarify the differences in timing and strength of the factors limiting growth at daily resolution during the growing season.

## Data Availability Statement

The raw data supporting the conclusions of this article will be made available by the authors, without undue reservation.

## Author Contributions

MVF and ASP designed this study and performed fieldwork. MVF measured and analyzed tree-ring width data. MVF, IIT, and VVS performed modeling. AVS contributed to the valuable comments and discussion. All authors wrote the manuscript.

## Conflict of Interest

The authors declare that the research was conducted in the absence of any commercial or financial relationships that could be construed as a potential conflict of interest.

## Publisher’s Note

All claims expressed in this article are solely those of the authors and do not necessarily represent those of their affiliated organizations, or those of the publisher, the editors and the reviewers. Any product that may be evaluated in this article, or claim that may be made by its manufacturer, is not guaranteed or endorsed by the publisher.

## Footnotes

1
http://www.meteo.ru/

2
http://www.vs-genn.ru/
