# Erratum: Plant–soil–climate interaction in observed and simulated tree-radial growth dynamics of downy birch in permafrost

**DOI:** 10.3389/fpls.2023.1185346

**Published:** 2023-03-23

**Authors:** Frontiers Production Office

**Affiliations:** Frontiers Media SA, Lausanne, Switzerland

**Keywords:** seasonal and annual tree-growth dynamic, temperature, precipitation, process based Vaganov-Shashkin model, VS-oscilloscope

An omission to the funding section of the original article was made in error. The following sentence has been added: “Open access funding was provided by the WSL - Swiss Federal Institute For Forest, Snow And Landscape Research”.

The original version of this article has been updated.

